# Balancing media selections over time: Emotional valence, informational content, and time intervals of use

**DOI:** 10.1016/j.heliyon.2023.e22816

**Published:** 2023-11-24

**Authors:** Mu-Jung Cho, Byron Reeves, Nilam Ram, Thomas N. Robinson

**Affiliations:** a Research Center for Humanities and Social Sciences, Academia Sinica, Taiwan; b Department of Communication, Stanford University, USA; c Department of Psychology, Stanford University, USA; d Department of Pediatrics, Stanford University, USA; e Department of Medicine, Stanford University, USA

**Keywords:** Media sequencing, Media selection, Psychological regulation, Selective exposure, Smartphone use

## Abstract

The sequencing of information in media can influence processing of content via mechanisms like framing, mood management, and emotion regulation. This study examined three kinds of media sequences on smartphones: (1) balancing positive and negative emotional content; (2) balancing emotional content with informational content; and (3) balancing time spent on and off the media device. Actual media use was measured in natural settings using the Screenomics framework which gathers screenshots from smartphones every 5 s when devices are on. Time-series analyses of 223,531 smartphone sessions recorded from 94 participants showed that emotionally positive content was more likely to follow negative content, and that emotionally negative content was more likely to follow positive content; emotional content was more likely to follow informational content, and informational content was more likely to follow emotional content; and longer smartphone sessions were more likely to follow longer periods of non-use.

## 1 Introduction

In an increasingly fragmented digital world where media experiences are woven from threads of radically different material, any message of interest—commercials, social media posts, online videos, text messages—is almost always surrounded by other media. Conventionally, different categories of media content are studied separately (e.g., studies about genres like advertising, news, social media) in their intended order (e.g., the parts of a narrative in a dramatic story). However, new interface affordances enable people to easily fragment and reorder material that was meant to be processed consecutively. The resulting content sequences may differ substantially from the intention of the communication sources as each user combines seemingly unrelated material in ways that make sense for them. Through the lens of psychological regulatory and balancing mechanisms, this study explores the individual creation of sequences that are created for both thoughtful or casual reasons.

Most media psychology research aims to eliminate spurious effects by separating target messages from their context. Examples are numerous: health communication studies have participants watch designated advertisements to investigate smokers’ message processing [1], behavior change game studies have people play one particular game to assess changes in targeted behaviors [2], virtual reality studies have research subjects interact with specific avatars to investigate the effect of embodiment on behavioral changes [3], and social media studies have participants view particular Instagram posts to evaluate the effect of social comparison [4]. This separation strategy is good for attributing effects; however, isolating messages from their natural media context is also risky. Several areas of research have demonstrated that message effects are often defined by what precedes and follows target messages as much as by the content of the messages themselves. For example, prospect theory [5] and the theory of attribution biases [6] are both supported by extensive empirical research on the contextual effects of information on attitudinal change, social behavior, and decision-making. A long history of media research has studied media context and its effect on message processing, from the early persuasion studies on primacy and recency effects [7] and priming effects [8], to the more recent work on television program context effects [9] and the theory of dynamic media processing [10]. These areas of research all demonstrate the importance of studying message *sequences* rather than effects of target segments taken out of context.

The importance of message sequences is growing as interactive technology matures. With greater user control, people fragment media experiences in any manner they choose and recombine message segments into threads of experience that rapidly weave in and out of numerous content categories [11]. Theories about media sequences are undermined when the objects of theory—the duration and qualities of media experiences—are changing quickly. Research paradigms that experimentally control the presentation of single message segments out of context, thus, have limited generalizability in a world where traditional media boundaries no longer apply [12]. We believe that research about moment-by-moment details of digital experiences and their sequences is central to media psychology. Theories of media psychology need grounding in new details about interactive media experiences, with particular emphasis on smartphone use. The pervasive and mobile nature of smartphones has resulted in significant transformations in people's digital lives. Analysis of the details of message sequencing would help researchers better understand the context of media experiences and create more grounded theories about the processing of media sequences.

This study is about three different message sequences that people experience when using smartphones—the sequence of positive or negative emotional content, the sequence of emotional or informational content, and the sequence to continue or stop smartphone use. Data were collected from actual smartphone use in natural environments and in a manner that preserved the sequences in which all the information was engaged. To observe the individual sequencing patterns at this fine temporal granularity, this study utilized an innovative research framework—Screenomics—to see how people manage media use at different timescales, and in particular moment-by-moment switching [11]. The Screenomics framework collects screens every 5 s that smartphones are activated, with the resulting recordings constituting a sequence of screen that are timestamped, and that can be analyzed individually (as representative of a 5-s epoch) or combined into different bundles that represent longer time segments of interest (e.g., the experience of a 1-min message, total screens by hours of the day or days of the week).

## 2 Balancing media experience through media content selection

### 2.1 Significance of research on media sequencing

Media sequences are defined as the temporal ordering of messages. A basic assumption for studying media sequencing is that messages are processed differently depending on their order and context. There are several pertinent literatures, often reviewed separately, that describe the influence of ordering on media experiences. Early work on primacy and recency effects on attitude formation and persuasion exemplifies sequence effects with respect to a message's position within a list of messages. Asch's [13] groundbreaking experiment demonstrated that reading a list of personality qualities in different orders can result in different personality impressions. The psychological research on primacy and recency effects influenced later marketing and advertising research that examined presentation order for commercial messages. For instance, Buda and Zhang [14] identified a recency effect of source credibility on the perceived attractiveness of and willingness to purchase a product. Recency effects are also found in the recall of television commercials [15].

The concepts of priming and framing in media effects research are also influences of sequence. Priming research proposes that exposure to messages may increase the salience of certain issues and ideas, which subsequently changes how a later message will be evaluated. Research on priming effects in political communication concerns how people change their standards for making political evaluations [16]. For instance, priming research has examined the effects of media campaign coverage on voter decisions, showing that campaign voters base their decisions on central campaign issues [17]. Framing research addresses the question of how people understand a message by comparing it with preexisting schemata [18]. Framing research emphasizes the importance of exposure to previous messages for interpreting later messages. For example, Iyengar [19] found that the episodic news frame, which depicts issues using specific instances, causes news viewers to attribute the responsibility for poverty more to individuals than society at large.

Newer forms of sequencing result from the active reconfiguration of information by users. Conventionally, effects of media sequences are studied as artifacts of media production; for example, the progression of scenes in a movie [20] or randomly ordered messages that occur by chance, such as the ordering of advertisements in a pod of television commercials [21,22]. For newer media, people may sequence unrelated information that joins quite different media content largely through quick switching between windows and applications. The resulting sequences are idiosyncratic to the individuals that compose them, representing a significant challenge to research. This study attempts to meet this challenge by examining individuals’ unique sequencing rather than sequences of content established by information producers.

The objective of this study is to bridge the gap between existing theories about media sequencing and the newer forms of sequencing that have not been thoroughly examined. We explore sequence effects through the lens of psychological regulatory and balancing mechanisms. Theories about regulatory mechanisms describe sequential processes in which individuals adjust emotions, manage cognitive resources, and make content choices according to preferences. Specifically, the study aims to examine whether smartphone users sequence media to regulate experiences between: (1) positive and negative content, (2) emotional and informational content, and (3) time spent on an off their smartphones.

### 2.2 Sequencing of positive and negative experiences

Considerable research has examined regulatory mechanisms that help individuals maintain balanced emotional states. Gross [23] describes a process model of emotion regulation. In this model, emotion regulation is a sequential process where people choose different regulation strategies at different stages of emotion generation. The stages are linked to attention and psychological appraisal of situations. For example, according to this model, a person may generate unpleasant emotions as a result of watching a horror movie. To regulate these unpleasant emotions, this person may choose to employ strategies to modify the situation, such as watching this movie with friends, or to change the appraisal of the situation, such as reminding oneself that it is only a movie.

The most pertinent aspects of emotion regulation to the present study are that people could increase, maintain, or decrease negative and positive emotions using strategies of psychological regulation [24]. Importantly, this process applies to the regulation of both positive and negative emotions. Down-regulation strategies (i.e., strategies for decreasing or minimizing the intensity of emotional experience) [25] for positive emotion may seem counter-intuitive from a hedonistic perspective, but in certain situations, a decrease of positive emotion may be beneficial. One explanation for decreasing positive emotions may be instrumental. Tamir [26] has argued that individuals may want to feel useful emotions, even if they are unpleasant, when future benefits are greater than immediate ones. Also, when emotion regulation is oriented to a goal, task, or norm, pain can be more desirable than pleasure [27]; for example, when people are expecting to interact with strangers, they may want to appear to be “cool and collected” in preparation for social interaction and regulate against their current mood state [28].

Another theory relevant to the sequencing of media experiences is the *counter-regulation principle* of automatic affective processing, which is defined as “the basic affective-motivational mechanism that allows an organism to remain in a state of a balanced sensitivity for gains and losses” [29]. The counter-regulation principle suggests that attention is automatically allocated to stimuli of opposite valence compared with current emotional and motivational states to maintain a balanced sensitivity [29]. Studies in affective research have indicated that positive or negative events usually have only short-term effects on emotional states [30,31], which supports the principle of counter-regulation. Experimental studies have provided direct evidence of attentional bias to emotional stimuli that are opposed to current emotional states [32,33]. The counter-regulation principle supports the perspective of balanced information processing; that is, sensitivity to both positive and negative information is an indispensable adaptive function. While mitigating negative emotion by attending to positive stimuli helps organisms to find solutions to aversive situations, attending to negative stimuli also allows organisms to identify potential danger and subvert impulsive actions [33]. Attentional counter-regulation promotes a balanced receptiveness to positive and negative information [27], resulting in the balanced processing of information [29].

Importantly, as a type of *automatic* emotional regulation, the counter-regulation principle describes an implicit, nonconscious process [29]. A prominent feature of digital media experiences may further emphasize unconscious regulation. Digital experiences are increasingly short, with most segments lasting only seconds. This means that many, and likely the majority of media segments are not long enough to allow thoughtful elaborations of how one is feeling so that a subsequent—and conscious—decision could be made about a new experience that would offer balance. More likely, the balancing is done quickly without a conscious or “slow thinking” rationalization [5].

Based on the emotion regulation research, a question about media sequencing is whether media selection varies between positive and negative content, as defined by the valence of the emotion corresponding to the content [12]. Is there evidence that individuals are engaged in a balancing mechanism as they move through positive and negative media content on smartphones? Based on the theory of emotion regulation, media selections may be considered strategies that aim to increase, decrease, or maintain emotions. Emotion regulation through media selections can be thought of as a situational selection or modification strategy through which individuals select or alter their situations to facilitate certain emotions or to change the emotional impact of a situation [23].

In media research, the concept of emotion regulation is primarily studied through the lenses of mood management theory, which proposes that media selections (and usually the selection of longer media experiences) are motivated by individuals’ mood states ([34]; also see review in Section 2.3). Empirical research has demonstrated that mood management may explain media selection behaviors with respect to television [35], music [36,37], social media [38,39], and computer games [40].

Experimental research has contributed to the validation of the counter-regulation principle. For example, Zhang et al. [41] demonstrated the effect of emotion counter-regulation by using video clips as prompts for assessing response time to pictures of happy and angry faces. Using winning or losing money as the stimuli for positive or negative outcome anticipation, Rothermund et al. [42] showed the attentional bias in the response time to positive and negative stimuli on a computer screen. Sassenberg et al. [43] prompted individuals to be sensitive to gains using memory tasks and demonstrated the attentional bias toward negative words. Greving et al. [44] examined attention to web pages, links and information and showed the attentional bias toward positive information in the condition which individuals felt threatened. According to the counter-regulation principle used to explain these results, to achieve balanced processing of media, one would expect smartphone users to select sequentially between positive and negative content. Thus, we posit that sequences of media selections on smartphones may show traces of regulatory strategies to achieve balanced emotional states. If the counter-regulation principle applies in the case of fast-paced smartphone sequences, then these sequences are results of automatic attention allocation without conscious intention in selecting balancing media content. Our first hypothesis about the balancing mechanism of media selections on smartphones proposes that we will find evidence of such regulation.

H1 Smartphone users balance their media content selection, with exposure to positive content more likely followed by negative content, and exposure to negative content more likely followed by positive content.

### 2.3 Sequencing of emotional and informational experiences

Research also suggests that individuals will balance emotional and informational experiences. In media psychology, Zillmann's three-factor theory of emotion is the foundation for several theories of media processes and effects, including excitation transfer and mood management theories. This theory describes emotion as the interaction of three principal components: the dispositional component that guides behavioral responses to emotion, the excitatory component that energizes behavioral responses to emotion, and the experiential component that is the conscious element of emotional experiences [45].

Based on Zillmann's definition of emotion, the theory of mood management draws on psychological theories about selective exposure [46] to explain how emotion may affect media use. Zillmann said that the basic thesis of mood management theory is that “consumption of messages—entertaining messages in particular—is capable of altering prevailing mood states, and that the selection of specific messages for consumption often serves the regulation of mood states.” ([47], p. 327) Mood management theory hypothesizes that media content is capable of altering mood states via excitatory potential and hedonic valence [47]. One of the most important hypotheses of mood management theory is about hedonistic consumption choices [47], which states that individuals tend to arrange stimulus environments to reduce negative mood state and maintain positive ones. This hypothesis has been both supported and challenged by empirical research. According to this hypothesis, individuals who are in negative mood states would prefer positive media to improve their mood. For individuals that are in positive mood states, although this tendency would be less strong, they would still prefer media that help them maintain a positive mood.

The hypothesis of hedonistic consumption choices has been corroborated in several studies. In one experiment, Knobloch and Zillmann [48] showed that individuals who were in negative mood states selectively exposed themselves to joyful music for longer periods of time. Another study used Ecological Momentary Assessment (EMA) methods to show that adolescents sustained positive mood states by consuming fun media [49]. Other studies have found mixed results about consumption choices. In contrast to the prediction of mood management theory, Knobloch et al. [50] found that individuals who are romantically in negative mood states preferred love-lamenting pop music. Two studies about gender differences in mood management also reported results inconsistent with theoretical expectations. They found that females showed a stronger preference than males for mood incongruent news [51] and music [37]. In both studies, male participants conformed less well with predictions of the original mood management theory.

The inconsistency between theoretical expectations and empirical findings led researchers to propose extensions to the original mood management theory. To account for non-hedonistic media choices, Zillmann [52] discussed several concepts that may help explain conflicting findings. Social comparison processes, for example, could be the reason that lonely individuals prefer media featuring similarly or more lonely individuals [54]. The concept of telic hedonism, which refers to the acceptance of bad moods in pursuit of good moods at a later time, may explain why the inhibition of good moods can be desirable when long-term gratification is anticipated. Among the various proposed extensions to mood management theory, the concept of informational utility is perhaps the most prominent. This conceptual extension acknowledges that informational need is a primary driving force of selective exposure to media. Zillmann [53] argued that there are two main determinants of media choices: hedonistic motivation dominates spontaneous choices for entertainment, and informational utility dictates the selection of educational and informational content. Zillmann's argument was rooted in Atkin's important differentiation between guidance-oriented and reinforcement-oriented selective exposure [55]. Zillmann [53] associated guidance-oriented selective exposure with informational utility, and discussed how media choices intended for learning and satisfying curiosity may explain findings that confounded the original mood management theory.

Zillmann's theory of selective exposure through media choices provides researchers with a useful framework to study balancing mechanisms that operate using media choices. Under this two-factor framework, media choices are either emotion-motivated or driven by the force of informational utility. From the perspective of regulatory theories, the complementary relationship between emotional and informational determinants of media choices suggests a potential balancing mechanism. In Atkin's terms, guidance- and reinforcement-oriented selective exposure each provides its kind of utility [55]. Utility, as a scientific concept, implies that media consumption has decreasing marginal benefits, i.e., each additional unit of consumed media content yields increasingly less utility, because the satisfaction of consuming each additional unit of media content diminishes as the media consumer gets more and more satisfied. The law of diminishing marginal utility is a well-studied theory and finds many applications in psychology [56,57]. If the law of diminishing marginal utility holds for both media selection driven by emotional and informational motivations, the utility yielded from selecting media content for one motivation will eventually be surpassed by the other type of motivation as selection processes unfold. This notion of sequential media selection based on diminishing marginal utility suggests how regulation may unfold over time. Research on psychological regulation underscores the adaptive value of variation and the adaptive functions associated with mood states and behaviors [58]. The variation between motivations for media selection allows media users to maximize their utility adaptively by alternating the type of content that is used for regulation. It is important to note that Zillmann's theory about media choices through regulation emphasizes also the automatic and unconscious nature of regulatory processes [53]. In the context of smartphone experiences, this theory would lead to an expectation that the fast sequencing of emotional and informational content occurs automatically without deliberative efforts.

Our second research question is about the sequence effect of media selections between emotional and informational content on smartphones. Here, in accordance with Zillmann's theoretical framework, emotional and informational content is defined by the emotional or informational qualities of the content that motivate media selection [52]. If such a balancing mechanism exists, we should be able to identify a sequence effect such that an increase in emotional media experience leads to an increase in the likelihood of experiencing informational content at a proximal point in time. Similarly, the sequence effect from the other direction should hold. We propose the following hypothesis.

H2 Smartphone users balance their media content selection, with exposure to emotional content more likely followed by informational content, and exposure to informational content more likely followed by emotional content.

### 2.4 Sequencing of times spent on and off smartphones

Time is fundamental to the characterization of media use patterns. In the case of smartphones, two temporal characteristics are elemental: the time when the smartphone is being used, and the time when it is not. These two states compose the on-off “rhythm” of smartphone use. A functional view of media use, primarily proposed by theories and research on the uses and gratifications of media, expects that individuals actively use media to fulfill goals and satisfy certain needs [59]. Past research on media use functions has defined many different functions (e.g., communication, entertainment, information seeking, and self-presentation) and how these functions are afforded by different media (blogs, music, social media, and television). A basic assumption of the theories of media use functions is that longer media use times and a higher number of uses indicate both stronger needs as well as a greater likelihood that needs can be satisfied [60,61]. If smartphone uses are driven by gratifications, then one would hypothesize that smartphone users are likely to turn off their devices for longer times after longer uses. This is because the likelihood to satisfy the goals of use and the extent to which users’ needs are gratified both increase as use time increases. Upon satisfaction of goals, users may be more likely to rest longer. Similarly, people are likely to use their smartphones for longer sessions after longer nonuse because needs have accumulated, and new goals have emerged.

Little research has been done on the sequencing of time spent on and off media at fast timescales (e.g., minutes or seconds), perhaps because of the difficulty in recording real-world media use at a sufficient level of granularity to reveal an on-off rhythm. For larger segments of media, survey-based research supports the idea that stronger needs are associated with more frequent uses of media and longer use times. Mehdizadeh [60] showed that individuals who have stronger needs for self-presentation check social media more frequently and spend more time in each session. Valenzuela et al. [62] found that the intensity of social media use, measured by user engagement and amount of time spent on media, is positively associated with the promotion of social capital. Chou & Liu [63] demonstrated that for middle-aged and elderly adults, the time and frequency of using a mobile messenger application is positively related to the amount of gratification received from the use. Chen [64] showed a positive relationship between the time users spent on a social media platform and their level of gratification for connection needs.

In sum, research has indicated that longer media use is associated with a higher likelihood of satisfaction. In the case of smartphone use, as the likelihood of satisfaction increases during longer smartphone interactions, there should be fewer needs to satisfy. As goals are fulfilled, the interactions with more media may lose novelty, engagement may wain, and the motivation to pursue more use will diminish. This may be true even for smartphone sessions that have no obvious goal. Device use that is casual and unfocused, but nevertheless engaging, will still draw down interest in sustaining use, and the reduced interest could then lead to longer rest or nonuse times. This may be true not only because there are no particular goals left to pursue but just because people get tired. Smartphone screens are compelling and designed to attract attention via auditory and pictorial novelty, including extremes in all aspects of presenting words and pictures. Engagement requires effort, and more effort requires longer breaks.

Our third hypothesis is about this sequence effect between use and nonuse.

H3 Smartphone users exhibit temporal balancing in their media use patterns, with longer smartphone use times more likely followed by longer periods of nonuse, and longer periods of nonuse more likely followed by longer use times.

## 3 The present study

The present study uses Screenomics [11] to examine sequences of media selection on smartphones that (1) balance positive with negative emotional content; (2) balance emotional content with informational content; (3) balance times spent on the device with time spent off the device. The study tests the three hypotheses about psychological regulatory and balancing mechanisms. H1 tests theories about emotion regulation and the counter-regulation principle. H2 tests theories about selective exposure through media selection motivated by emotional and informational utilities. H3 tests basic assumptions of the functional theories of media use.

Screenomics involves a data collection and analysis protocol that facilitates the study of individuals' psychological experiences on digital devices *in situ* [11]. Screenshot images are collected unobtrusively from individuals’ smartphones every 5 s when device screens are turned on. The resulting screenshot sequences, *screenomes*, are granular-level records of all screen content and behavior, across all different apps, software, and platforms—whatever is on a screen. The sequential structure of the data preserves the psychological context of media experience, including meta-data about the physical location in which the screen was viewed while allowing for flexible sampling strategies and analysis across multiple timescales. Here, Screenomics is used to observe the media selections that precede and follow specific types of content and behavior. Considering the literature on which our three hypotheses were based, an important acknowledgement is that audio content of smartphone use cannot be analyzed with the current research design. While Screenomics permits research on visual content, the present study focused solely on texts extracted from screenome. Theories and research concerning emotion regulation, mood management, and functions of media use all underscore the importance of audio and visual content on media choices.

## 4 Methods

### 4.1 Participants and procedure

Data were drawn from the Human Screenome Project (see also [11,[65], [66], [67]]). Data collection was approved by the IRB of the authors' institution (protocol number: 38485). Participants were 178 adults (18 or more years of age) recruited via a commercial research firm from focus group sessions of smartphone users in Chicago, Los Angeles, and New York. After expressing interest in this study of mobile device use, participants were given additional information about the goals of the study, the data collection protocol, and the potential risks and benefits of their participation. Those who agreed to participate and provided informed consent were asked to use their devices as usual during the next weeks while software that was downloaded onto their devices unobtrusively collected screenshots of whatever appeared on their device's screen. Over the next few weeks, screenshots were collected every 5 s through a privacy-preserving protocol where screenshots were automatically encrypted and transmitted to a secure research server for later processing and analysis. The 5-s interval was used so that the data permit analysis of media content and use behavior at a much faster timescale than what had been done in prior research, hence opening a new realm of behavior to scientific inquiry. Practically, in our testing of the Screenomics data collection protocol, an even shorter interval may occasionally burden participants' smartphones and affect their smartphone use. At the end of the collection period, the software was removed from the devices, and the participants were thanked and given compensation ($100 for the focus group, $200 for 10 days of Screenomics data collection, and an additional $100 for one month of Screenomics data collection).

The present analysis uses data provided by *n* = 94 of the 153 individuals who enrolled in the follow-on study and who successfully provided more than 100 screenshots over at least 7 consecutive days. The 100-screenshot cutoff translates to a minimum of 9 sessions (the time between turning on and turning off the screen) per day in the data. This allowed for examination of temporal patterns while maintaining a balance between the length of time-series and the comprehensiveness of temporal coverage. The average number of sessions across person-days is 340.6 (*SD* = 261.77, median = 277, range = 9 to 1,629, IQR = 249). These 94 participants (46 female, 47 male) self-identified as Caucasian (*n* = 51), Hispanic (*n* = 20), African American (*n* = 15), and Asian American (*n* = 8), and were age <26 (*n* = 18), 26 to 32 (*n* = 21), 33 to 37 (*n* = 21), 38 to 42 (*n* = 11), or > 42 years (*n* = 23). The study analysis uses screenshots obtained during the first 7-days of collection, a total of 1,585,668 screenshots obtained during 223,531 unique smartphone sessions. Table 1 shows the distribution of the numbers of daily and total sessions across participants.

**Table 1:** Distributions of the numbers of daily and total sessions over participants.

	Mean	Median	SD	IQR	Range
Daily sessions	340	286.64	233.62	186.07	54.71–1236.29
Total sessions	2379.99	2006.5	1635.33	1302.5	383–8654

### 4.2 Measures

Screenshots were measured and processed using a pipeline that extracted a variety of temporal, textual, graphical, and topical features from each screenshot (see Refs. [11,68,69] for details). In brief, textual and graphical information embedded in each screenshot was extracted using custom modules wrapped around OpenCV [70] and Tesseract-based [71] optical character recognition (OCR) tools. Basic graphical features, including graphical entropy, number of faces detected, probability of logo presence (e.g., Facebook, Instagram, CNN, ABC), presence of a touchscreen keyboard, were complemented by text-based character-level features (e.g., number of characters, ratios of alphanumeric characters, counts of punctuation marks), and lexicon-based features that were derived after non-alphabetic characters, single-character words, and extra whitespaces were removed. The remaining words were spell-checked using Hunspell [72] and compared to a token list that retained specific words (e.g., “cybersocial,” the name of our data collection app). Specific psycholinguistic features were summarized using the Linguistic Inquiry and Word Count (LIWC; [73]) and custom dictionaries (e.g., Facebook terms like “comment”, “like”, “post”, “react”). Altogether, there were 128 textual and graphical features extracted for each screenshot and used in subsequent analyses as detailed below. R was used for data analysis.

***Session length and Gap*.** Temporal features of each individuals’ smartphone use and non-use were calculated with respect to smartphone *sessions*—defined as the sequence of continuous screenshots obtained between screen on and screen off events (i.e., screenshots that are not more than 5 s apart). Session *length* was calculated for each session as the amount of time that the smartphone was on continuously. Session *gap* was calculated for each session as the amount of time elapsed since the end of the previous session. Across participants, the average session length was 35.38 s (*SD* = 128.62, median = 10, range = 5 to 16,070, median absolute deviation = 7.413), and the average gap between sessions was 212.85 s (*SD* = 1673.33, median = 15, range = 10 to 146,080, median absolute deviation = 7.413).

***Positivity and Negativity.*** The emotional content of each session was measured using the LIWC textual features [73]. Specifically, *positivity* of each session was calculated as the average of the percent of positive words (*posemo*) identified in each screenshot in the session. Similarly, *negativity* of each session was calculated as the average of the percent of negative words (*negemo*) identified in each screenshot. Words that constitute the *posemo* and *negemo* categories of the LIWC dictionary were derived from a series of studies for the identification of word categories that constitute common emotional and cognitive dimensions of psychology research [73]. For both scores, higher values indicate more emotional content. Across participants, the average session had positivity of 2.33 % (*SD* = 3.66, median = 0.55, range 0–100, median absolute deviation = 0.81) and negativity of 0.93 % (*SD* = 3.15, median = 0, range = 0 to 100, median absolute deviation = 0).

***Factuality.*** Using a combination of manually coded data and machine learning, each screenshot was classified with respect to *factualit*y of content, specifically defined as the likelihood that screen content contained verifiable claims, a definition that taps into Zillmann's concept of the informational utility that drives media choices [52]. Regardless of content type, factuality measures informational utility that contrasts the utility of emotional content. Two research team members (undergraduate research assistants authorized to examine the data) were trained to label factual vs. non-factual screenshots (binary classification) and achieved a 90 % level of agreement on a set of 3069 screenshots. These team members then labeled a randomly selected set of 47,415 screenshots. These ground truth data, and the textual and graphical features described above, were then used to train a machine learning model that propagated labels to the rest of the data. Of the several models tested and tuned using grid or random search across repeated cross-validation samples (including naive Bayes, support vector machine, random forest), an eXtreme Gradient Boosting (XGBoost; [74]) model provided the best accuracy, 0.9847, a Cohen's kappa of .8291, and a F1 score of 0.9908. Using this model, each screenshot was given a factuality score (softmax probability). Factuality scores were then calculated for each session as the average of the screenshot scores. Higher values indicate a greater presence of verifiable informational content, based on both the textual and graphical features of the screens. Across participants, the average session had factuality of 0.1 (*SD* = 0.2, median = 0.01, range = 0 to 1).

Table 2 presents the correlation matrix for the session-level variables, including the lag-1 variables of *Factuality*, *Positivity*, and *Negativity*. Table 2 indicates that the session-level variables are not linearly correlated and that screen that is rich in informational content is not also rich in affective content. The weak correlations between informational content and affective content suggest that lag-1 affective content may influence flow if informational content. The correlation between *Positivity* and *Negativity* indicates that positive and negative content mostly appear on different screens, rather than on the same, affectively rich screens. However, the weak correlation between *Positivity* and *Negativity* suggests that there is some complexity in both the temporal inertia of *Positivity* and *Negativity* and how they influence each other over time.

**Table 2:** Correlation matrix of the session-level variables.

	Factuality	Positivity	Negativity	Length	Gap	Factuality (lag-1)	Positivity (lag-1)	Negativity (lag-1)
Mean (*SD*)	9.90 (*20.17*)	2.33 (*3.66*)	0.93 (*3.15*)	35.38 (*128.62*)	212.85 (*1673.33*)			
Factuality	1							
Positivity	.058	1						
Negativity	.035	.042	1					
Length	.032	.048	.014	1				
Gap	−.026	−.018	.013	.012	1			
Factuality (lag-1)	.701	.059	.035	.011	−.021	1		
Positivity (lag-1)	.056	.495	.037	.036	−.008	.058	1	
Negativity (lag-1)	.032	.031	.531	.004	.016	.035	.042	1

Total number of sessions: 223,531.

## 5 Data analysis

**Fig. 1:**
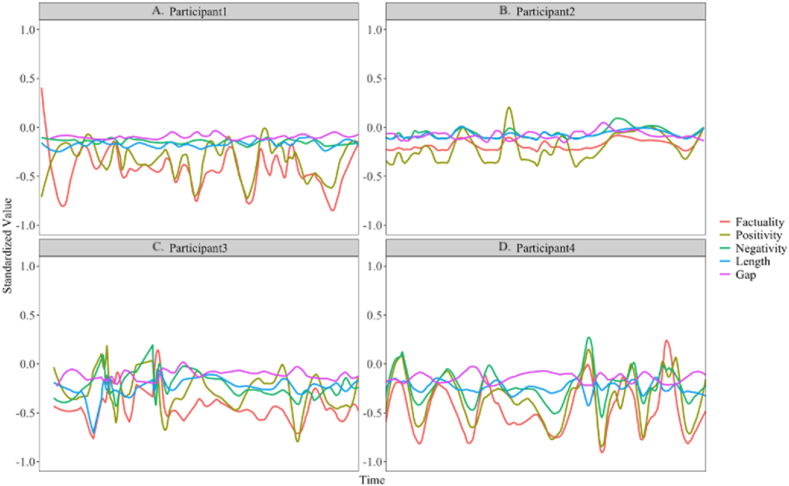
Typical daily trends of positivity, negativity, and factuality, session length, and gap.

Standardized data on the session-to-session progression of session length, session gap, positivity, negativity, and factuality of four participants' week-long time series are shown in Fig. 1. Using these multivariate time-series, the session-to-session dynamics of all 94 participants' media selections (including the 4 shown in Fig. 1) were examined using a multilevel vector autoregression (mlVAR) model that accommodated the nested nature of the data (sessions nested within persons) while prioritizing examination of the within-person relations among the five variables used to describe individuals' media selections. Following implementations used in analyses of similarly structured experience sampling data [75,76], the collection of 94 individuals’ session-to-session time-series data were modeled as

(1) $$Y_{t}^{\left(p\right)}|y_{t}^{\left(p\right)}=\mu^{\left(p\right)}+{Β}^{\left(p\right)}\left(y_{t-1}^{\left(p\right)}-\mu^{\left(p\right)}\right)+\varepsilon_{t}^{\left(p\right)}$$

where the standardized multivariate time series for person *p* at time *t*, that is conditioned on the value of the other variables at time *t*, $Y_{t}^{\left(p\right)}|y_{t}^{\left(p\right)}$, is described using $\mu^{\left(p\right)}$, a person-specific vector of intercepts that encodes expected means for the 5 variables, ${Β}^{\left(p\right)}$, a person-specific matrix of regression coefficients that encode the temporal relations among the 5 variables, and $\varepsilon_{t}^{\left(p\right)}$, a multivariate time-series of residuals that have variance-covariance matrix Θ that encodes (in inverse form) the contemporaneous relations among the 5 variables. In turn, between-person differences in the expected means and temporal relations are modeled as

(2) $$\mu^{\left(p\right)}=\mu^{\left(base\right)}+m^{\left(p\right)}$$

and

(3) $${Β}^{\left(p\right)}=\beta^{\left(base\right)}+b^{\left(p\right)}$$

where $\mu^{\left(base\right)}$ and $\beta^{\left(base\right)}$ are vectors of fixed effects that encode the means and temporal relations for the average person, and $m^{\left(p\right)}$ and $b^{\left(p\right)}$ are vectors of random effects that have a variance-covariance matrix Ω that encodes the between-person relations. The model was fit using the mlVAR package in R [77]. Of specific interest for our research questions is the vector of fixed effects $\beta^{\left(base\right)}$ that encode the session-to-session temporal dynamics of the 5 variables for the prototypical person in the sample. Post-fitting examinations indicated that the residuals of all five variables were acceptably normal with relatively similar misfit (in opposite directions) at both low and high ends of the range.

## 6 Results

**Fig. 2:**
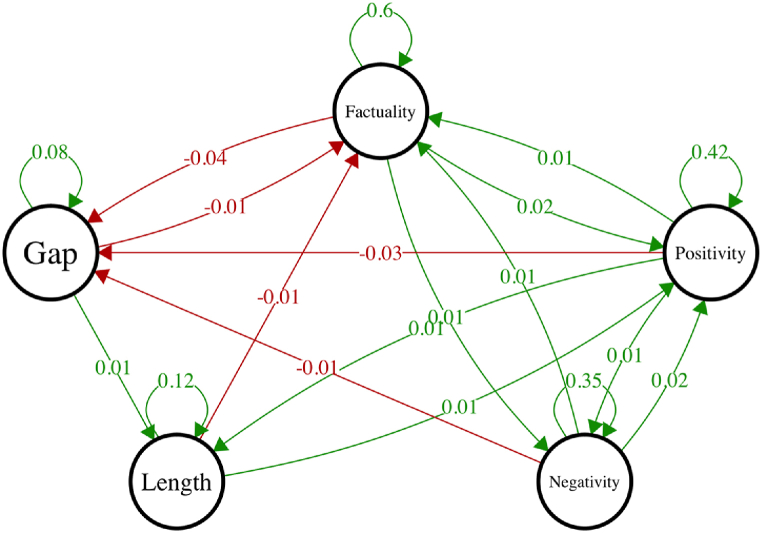
Within-person temporal (lag-1) network of the multilevel vector autoregression model. The direction of the effect from *t* to *t+1* is indicated by arrows. Green (red) arrows indicate positive (negative) effects. Only significant effects are included (*alpha* = 0.05). (For interpretation of the references to colour in this figure legend, the reader is referred to the Web version of this article.)

The multilevel VAR model was used to capture and articulate how the characteristics of any given smartphone session influenced participants’ behavior during the next session. Parameter estimates describing these dynamics are shown in Table 3 (temporal relations of main interest), with graphical representations of the temporal relations relevant for the hypotheses shown in Fig. 2 (with other aspects of the model shown in Tables S1 and S2, and Figs. S1 and S2, in the supplement). Only significant effects (*alpha* = 0.05) are included in the figure. Green and red arrows and numbers indicate direction and standardized effect size of temporal relations for the prototypical person. For example, the green arrow at the top right of Fig. 2 indicates that when *positivity* of a session (*t*) increased by 1 SD, the *factuality* of the subsequent session (*t* + 1) was expected to increase by *β*_*positivity → factuality*_ = 0.01 SD. Similarly, the red arrow at the top left of the figure indicates that when *factuality* increases by 1 SD, the *gap* to the next session would shorten by *β*_*factuality*__→__*gap*_ = −0.04 SD. Simultaneous dynamics indicated by the temporal network include, for example, that when *factuality* increased by 1 SD, the next session is likely to have both higher positivity (*β*_*factuality → positivity*_ = 0.016) and higher negativity (*β*_*factuality → negativity*_ = 0.011). Specific sets of parameters inform the hypotheses.

**Table 3:** Temporal (lag-1) effects of the multilevel vector autoregression model.

From	To	Fixed effect	SE	p-value	Random SD
Factuality	Factuality	0.596	0.013	<0.001	0.117
Factuality	Positivity	0.016	0.007	0.003	0.045
Factuality	Negativity	0.011	0.007	<0.001	0.021
Factuality	Length	0.011	0.005	0.064	0.05
Factuality	Gap	−0.036	0.005	<0.001	0.03
Positivity	Factuality	0.012	0.003	<0.001	0.024
Positivity	Positivity	0.417	0.016	<0.001	0.138
Positivity	Negativity	0.010	0.004	0.003	0.025
Positivity	Length	0.014	0.004	0.001	0.031
Positivity	Gap	−0.032	0.003	<0.001	0.038
Negativity	Factuality	0.007	0.005	0.009	0.017
Negativity	Positivity	0.016	0.004	<0.001	0.021
Negativity	Negativity	0.347	0.017	<0.001	0.16
Negativity	Length	0.004	0.002	0.394	0.033
Negativity	Gap	−0.012	0.004	0.004	0.033
Length	Factuality	−0.013	0.003	<0.001	0.024
Length	Positivity	0.010	0.003	0.031	0.038
Length	Negativity	−0.001	0.002	0.496	0.008
Length	Length	0.122	0.008	<0.001	0.067
Length	Gap	0.011	0.008	0.169	0.072
Gap	Factuality	−0.006	0.002	0.001	0.006
Gap	Positivity	−0.002	0.002	0.226	0.005
Gap	Negativity	0.022	0.007	0.074	0.119
Gap	Length	0.011	0.005	0.019	0.039
Gap	Gap	0.076	0.007	<0.001	0.069

*Sequencing of positive and negative experiences*. In line with hypothesis H1, results indicate that exposure to positive content increases the likelihood of subsequent engagement with negative content, (*β*_*positivity*__→__*negativity*_ = 0.01, *p* = .003); and that exposure to negative content increases the likelihood of subsequent engagement with positive content, (*β*_*negativity*__→__*positivity*_ = 0.016, *p* < .001). This feedback loop, wherein the positivity and negativity nodes continually influence each other over time, indicates the presence of an emotion balancing mechanism.

### 6.1 *Sequencing of emotional and informational experiences*. In line with the hypothesis

H2, results indicate that exposure to emotional content increases the likelihood of subsequent engagement with informational content, (*β*_*positivity*__→__*factuality*_ = 0.012, *p* < .001) and (*β*_*negativity*__→__*factuality*_ = 0.007, *p* = .009). In complement, exposure to informational content increases the likelihood of subsequent engagement with emotional content, (*β*_*factuality*__→__*positivity*_ = 0.016, *p* = .003) and (*β*_*factuality*__→__*negativity*_ = 0.011, *p* < .001). That is, exposure to emotional content increased the likelihood of engaging informational content, and exposure to informational content increased the likelihood of engaging emotional content. Here, the feedback loops embedded in the temporal relations indicate balancing of selections between emotional and informational content.

*Sequencing of times spent on and off smartphones*. Contrary to hypothesis H3, there was no evidence of a feedback loop between session length and session gap. Session gap was related to subsequent session length, (β_*gap*__→__*length*_ = 0.011, *p* = .02), such that the longer the gap the longer the subsequent session. However, session length was not related to the subsequent session gap (β_*length*__→__*gap*_ = 0.011, *p* = .17). This pattern of temporal relations lends only partial support to the hypothesis about rhythm in media use and non-use.

## 7 Discussion

### 7.1 Empirical and theoretical contributions

The present study examined three hypotheses about the sequencing of media selections. These hypotheses were built on psychological theories about regulatory mechanisms of emotion, mood management, selective exposure, and media functions. Our results demonstrate how smartphone users balance engagement with media. The findings suggest that users balanced positive sessions with negative ones, supporting the hypothesis about emotion regulation and the principle of counter-regulation. The findings also suggest that smartphone users balanced exposure to emotional and informational content, supporting the hypothesis rooted in the two-factor framework of selective exposure. And, results partially supported the hypothesis about the sequencing sessions of various lengths. Longer screen-off time was more likely to be followed by longer sessions, although longer sessions were not significantly more likely to be followed by longer off time. Altogether, the results inform knowledge of sequence effects in media and the psychological mechanisms that shape individuals’ engagements with media.

The most general implication of the results is that there is a rhythm to the progression of smartphone experiences that transcends the traditional categories of applications (e.g., Facebook, YouTube), content genres (e.g., news, entertainment), or content providers (e.g., New York Times, CNN). Within smartphone sessions—where people switch frequently among different applications and content—there is a cadence to media engagement that creates evenness in smartphone experiences. Smartphone experiences can be happy or sad, emotional or informational, and effortful or quick, but examined as a time course of experiences, the extremes of those dichotomies are balanced in a manner that tends toward homeostasis.

These results add to the literature about why and how people choose media experiences (e.g., Refs. [78,79]), but at a much more granular level of selections. The time domain of the sessions analyzed in this study, on average only 35.4 s long, shows that considerations of uses and gratifications apply importantly to the quickened pace of new technology. In addition to using smartphones to achieve particular goals (e.g., someone might say they use smartphones primarily to experience positivity), people appear to use particular short sequences to engage specific kinds of content, counterbalancing positive and negative content and emotional and informational content. This is added evidence that characterizations of media use should not only, and perhaps not even primarily, be attributed to devices or content genres *per se*. People use smartphones for a variety of reasons, and they appear to balance their use across sessions lasting only seconds. The implications of this finding for research design in media psychology are important. There is much to be learned from examination of media uses and effects with the kinds of fine-grained data that make the underlying psychological processes of interest visible—a clear push for intensive repeated measures designs that track individuals over time and deemphasis on cross-sectional designs that examine group differences at a single point in time (e.g., Refs. [80,81]).

There are additional sequencing theories that may explain individuals’ smartphone use, especially the cadence of engagements with informational and emotional content. A closer analysis of content engagements (not part of the hypotheses of this study) could show that much of the emotional content is obtained from engagements with entertainment (e.g., games, YouTube clips) and social interactions (e.g., conversations with friends in social media) while much of the informational content is obtained from engagements with more serious news stories (whether on news websites or social media posts) and domain-specific sites. This might suggest that, in addition to impulses to counterbalance emotional and informational content, people may also be regulating a sense of time well spent or time misused. There is evidence of such trade-offs in the area of moral self-licensing [82], for example, which shows that people are more likely to engage in behaviors that were unethical if they had recently performed good deeds. The positive behaviors were thought to give people “credits” that could be spent on problematic future behaviors. Consumption of factual content, perhaps especially if it is connected to civic responsibilities, educational endeavors or work, may similarly constitute psychological credits for more frivolous future interactions like gameplay or social gossip. And the reverse may be true as well; time that people think was not used well could increase the need to replenish their “credits” by turning to serious content. It is important to note that such trade-offs, if indeed applicable to our data, are likely occurring in the minutes between seconds-long sessions—suggesting that the impulses to change and shift among tasks and content can be accomplished quickly.

The speed of session changes that are captured in this study (e.g., the speed with which a positive emotional experience might increase the probability of a subsequent negative one), is also important for conclusions about an *accumulation* of sequence effects. The effects of any single switch were significant in our data but they were not substantial (e.g., the effects sizes ranged only from .01 to .04). The switching that completed the balancing, however, occurred between sessions that lasted on average *only* 35.4 s. Added to the average length of the gaps between sessions (212 s), this means that one switch to achieve balance occurred within a window of approximately 4 min. This short cycle allows for a large number of balancing sequences per day per person. The small effects are likely substantially magnified and more consequential, given that, on average, individuals are switching over 200 times per day [65].

Screenomics as applied in the present study also offers an opportunity to expand the theoretical explanations of sequencing in new directions. Of particular interest may be the application of dynamic systems theory [[83], [84], [85], [86], [87]]. Dynamic systems theory is chiefly concerned with the evolution of communication over time, its context which includes physical locations, and the interaction among nested systems. These nested systems comprise a variety of biological, psychological, and social layers. These are each integral to Screenomics analysis. Central to Screenomics is an idiographic research strategy that focuses on intraindividual changes over time, and that allows zooming in and out across timescales, from moments to weeks, months, and years. The ability to simultaneously examine behavior at multiple time-scales links well to dynamic systems theory. Behavior at each timescale can be linked to different system layers that all together influence how media is experienced. We are excited about the possibility to use these new data and methods to discover how systems (or control parameters) governing behaviors at different timescales interact and constrain each other.

### 7.2 Limitations and future directions

First, although the sample was somewhat demographically diverse, the use of a convenience sample of participants diminishes sample representativeness. Future research could compare our findings with results obtained with larger and more representative samples and also test whether the noted associations remain stationary over time. Second, we employed computational methods to extract textual and graphical features using natural language processing and machine learning. While these methods enable measurement at scale, caution is warranted, given issues of feature omission and possible measurement biases. For example, this study measured only textual emotional valence and omitted other emotional content, such as human faces in pictures, that may have also appeared on the screens. Future studies could incorporate additional content features and measure those variables more comprehensively.

A third limitation is the 5-s sampling interval. While this interval is extremely short compared to survey-based studies and other experimentally controlled experiences, it may still miss some aspects of media experiences that change quickly. We are already finding that our definitions of smartphone use and rest times are somewhat constrained by the 5-s interval. The sampling interval determines researchers' ability to examine the dynamics of individuals’ media experiences [88], and we were only able to observe rhythms that manifested at cadences longer than 5 s. We note many situations where people appear to engage with particular types of content for *less* than 5 s to satisfy gratifications or balance previous exposure; for example, a 1–2 s look at a new photo of a family member (positive experience) after reading a troubling news story (negative experience). Future research may consider using even shorter sampling intervals that can capture these potentially consequential micro-exposures.

Fourth, this study is also limited by our definition of factuality and its use to quantify informational utility. Our definition of factuality—the likelihood of having verifiable information on smartphones screens—is different from the definition used in the fact-checking literature [89]. We were able to obtain factuality scores, regardless of content type. Although this approach may miss some components of content that drive utility-based selective exposure, the measure provided a proxy of informational utility that usefully captured the contrast between informational and emotional content that manifested at the fast timescale and fine granularity of our data.

Fifth, there is concern that Screenomics is not able to directly examine the psychological mechanisms of processing but rather only the products of that processing that are apparent in the screens that are chosen and produced. One solution is to supplement screen data with periodic surveys (e.g., delivered via text messages on the devices that are collecting the screens) that ask participants to comment on screen sequences, and indicate thoughts and emotions experienced during processing. That method, however, is difficult to use when the major interest is in extremely short sequences that are quite difficult to remember and match with questions that people might be able to answer. The shortness of the screen sequences means that it will be difficult, at least in the wild outside of the lab, for people to offer introspective comments about fast-moving sequences when they are interrupted with questions. It is also worth noting that the speed at which screen experiences change also means that conscious psychological mechanisms that drive sequence experiences are proximal to the behaviors induced by the screen stimuli (i.e., making screen choices), so much so that it could be near impossible to gain any additional visibility into the fast interplay of thinking, feeling and behavioral choices without introducing cumbersome parallel collections of physical signals related to processing (e.g., Ref. [90]). Introspective commentary about screen choices, typically accomplished via ecological momentary assessments (EMA), could be labeled the ground truth about the psychology that drives media use. However, the opposite may be equally true. Without actual behaviors (i.e., alterations in screen choices and sequencing that screenomics uncovers), it is reasonable to question the validity of psychological assessments that have no behavioral counterpart.

Sixth, we would also like to acknowledge the limitations inherent in interpreting our findings from the perspective of user-driven media selections and an implied high level of user agency. There are situations where the sequences we observed could be products of factors external to the user. For example, situations where: (a) sessions were prompted by notifications (e.g., app reminders, text messages), (b) sequences of content were curated by websites or apps rather than the users (e.g., scrolling through news feed), (c) sequences were generated habitually and compulsorily, indicating a reduction in user control (e.g., compulsive social media use), and (d) sequences were created due to the tendency of users to return to similar content in subsequent sessions. The last situation raises a possibility. If content within the same session tend to be both affective and informational, or both negative and positive, then the lag-1 effects found in our analyses could simply be the result of autocorrelations. For instance, if sessions characterized by a substantial amount of positive content also tend to contain a significant amount of negative content, and if similar types of content tend to appear successively, it is possible to observe a pattern where negative content is more likely to follow positive content and vice versa. However, as seen in Table 2, the correlations between informational content and affective content, as well as between positive and negative content, are too weak to justify this alternative explanation. This is further evidenced by the contemporaneous network included in the supplementary materials, which shows that the correlations at the same time point are not substantial enough to fully account for the temporal effects discussed in this study. It is thus important to note that the range of possible situations will require more precise theorizing about the primary mechanisms driving user behavior, how factors external to the users influence and moderate user behavior, and additional clarity on assumptions that are baked into the theory.

Lastly, this study is limited by its analytical methods. The multilevel VAR analysis supports network representation of within-person temporal dynamics and contemporaneous associations, and between-person associations (results included in the supplement). The present study used only the within-person network to test our hypotheses of sequence effects. Future research can use the between-person associations to investigate theories about inter-individual differences in media selections, expand into the development and testing of theoretically derived hypotheses about the interactions among the dimensions examined in this study (e.g., are long and positive sessions followed by short and negative sessions?), and examine if and how the temporal relations may be driven by nonlinearities in particularly extreme instances of the behaviors. Additionally, this study analyzed only a small subset of the temporal structures of smartphone interactions. Sessions can be defined in ways different from this study's definition, for example, treating any windows of nonuse shorter than 10 s as continuing the same session. The temporal dynamics of smartphone interactivity is much broader than what has been examined in this study and is a promising direction for future research.

A promising avenue for future research, which this study did not fully explore, is the significant impact of small content markers. For instance, Kim et al. [91] discovered that the hashtag '#Ad' reduces message credibility on social media, leading to endorsements by human-like virtual influencers being perceived as no more credible than those by anime-like virtual influencers. This finding suggests that even minor content elements can drastically influence how media users respond to the overall content. More broadly, while this study identified certain psychological factors shaping the rhythm of smartphone use through sequencing mechanisms, many external cues remain unexplored. These include subtle content markers, notifications, content curation algorithms, and advertisements. Collectively, these factors present a multifaceted view of digital media experience, where various sequencing mechanisms influence each other across different timescales and content types. Future research should prioritize identifying these factors and assessing their influence on smartphone users' media experiences.

### 7.3 Conclusion

In conclusion, this study examined three kinds of balancing that may manifest in smartphone users' engagements with media experiences—and a set of hypotheses derived from media psychology theories about emotion regulation, selective exposure, and media functions. Using Screenomics to obtain fine-grained observation of individuals’ engagement with fast-moving digital media experiences and time-series methods that support articulation and study of temporal dynamics we examined session-to-session balancing of positive vs. negative content, emotional vs. informational content, and smartphone use and rest times.

The results provided support for the principle of counter-regulation, as evidenced by the trend of sessions becoming increasingly negative following emotionally positive sessions, and vice versa. Moreover, the results also aligned with the two-factor framework of selective exposure, demonstrating that sessions following emotional sessions tended to become more informational, while sessions following informational sessions tended to become more emotional. The results partially supported the hypothesis of media functions, longer screen off time was more likely to be followed by longer sessions although longer sessions were not significantly more likely to be followed by longer off time. These findings highlight that media use unfolds for individuals over time, and in a manner that offers people a chance to actively self-regulate experiences through sequenced engagement with positive vs. negative and emotional vs. informational content.

## Data Availability

The data associated with this study has not been deposited to a publicly available repository, due to it confidentiality.

## Ethics statement

This study was approved by the IRB of the authors’ institution (protocol number: 38485).

## CRediT authorship contribution statement

**Mu-Jung Cho:** Conceptualization, Data curation, Formal analysis, Investigation, Methodology, Project administration, Resources, Software, Supervision, Validation, Visualization, Writing - original draft, Writing - review & editing. **Byron Reeves:** Conceptualization, Data curation, Funding acquisition, Investigation, Methodology, Project administration, Resources, Supervision, Validation, Writing - original draft, Writing - review & editing. **Nilam Ram:** Conceptualization, Data curation, Formal analysis, Funding acquisition, Investigation, Methodology, Resources, Supervision, Validation, Writing - original draft, Writing - review & editing. **Thomas N. Robinson:** Conceptualization, Data curation, Funding acquisition, Investigation, Methodology, Resources, Supervision, Validation, Writing - original draft, Writing - review & editing.

## Declaration of competing interest

The authors declare the following financial interests/personal relationships which may be considered as potential competing interests:

Byron Reeves reports financial support was provided by John S and James L 10.13039/100005959Knight Foundation. If there are other authors, they declare that they have no known competing financial interests or personal relationships that could have appeared to influence the work reported in this paper.
