# Supplementary material for: Balancing media selections over time: Emotional valence, informational content, and time intervals of use
Source: Heliyon. 2023 Nov 24;9(12):e22816. doi: 10.1016/j.heliyon.2023.e22816 (PMC10731070; doi:10.1016/j.heliyon.2023.e22816)
Supplement: Multimedia component 1 [file mmc1.docx]

**Supplement**

| Measure 1 | Measure 2 | Partial Correlation | Random SD of  Partial Correlation |
| --- | --- | --- | --- |
| Positivity | Factuality | 0.016 | 0.003 |
| Negativity | Factuality | 0.016 | 0.001 |
| Negativity | Positivity | 0.035 | 0.003 |
| Length | Factuality | 0.052 | 0.009 |
| Length | Positivity | 0.062 | 0.006 |
| Length | Negativity | 0.026 | 0.001 |
| Gap | Factuality | -0.027 | 0 |
| Gap | Positivity | -0.013 | 0.001 |
| Gap | Negativity | 0.031 | 0.007 |
| Gap | Length | -0.001 | 0 |

**Table S1.** Contemporaneous effects of the multilevel vector autoregression model

| Measure 1 | Measure 2 | Partial Correlation | Correlation |
| --- | --- | --- | --- |
| Positivity | Factuality | 0.008 | 0.081 |
| Negativity | Factuality | 0.251 | 0.266 |
| Negativity | Positivity | 0.298 | 0.302 |
| Length | Factuality | -0.007 | 0.006 |
| Length | Positivity | 0.135 | 0.098 |
| Length | Negativity | -0.062 | -0.019 |
| Gap | Factuality | 0.068 | 0.072 |
| Gap | Positivity | -0.117 | -0.075 |
| Gap | Negativity | 0.044 | 0.023 |
| Gap | Length | 0.257 | 0.245 |

**Table S2.** Between-person effects of the multilevel vector autoregression model


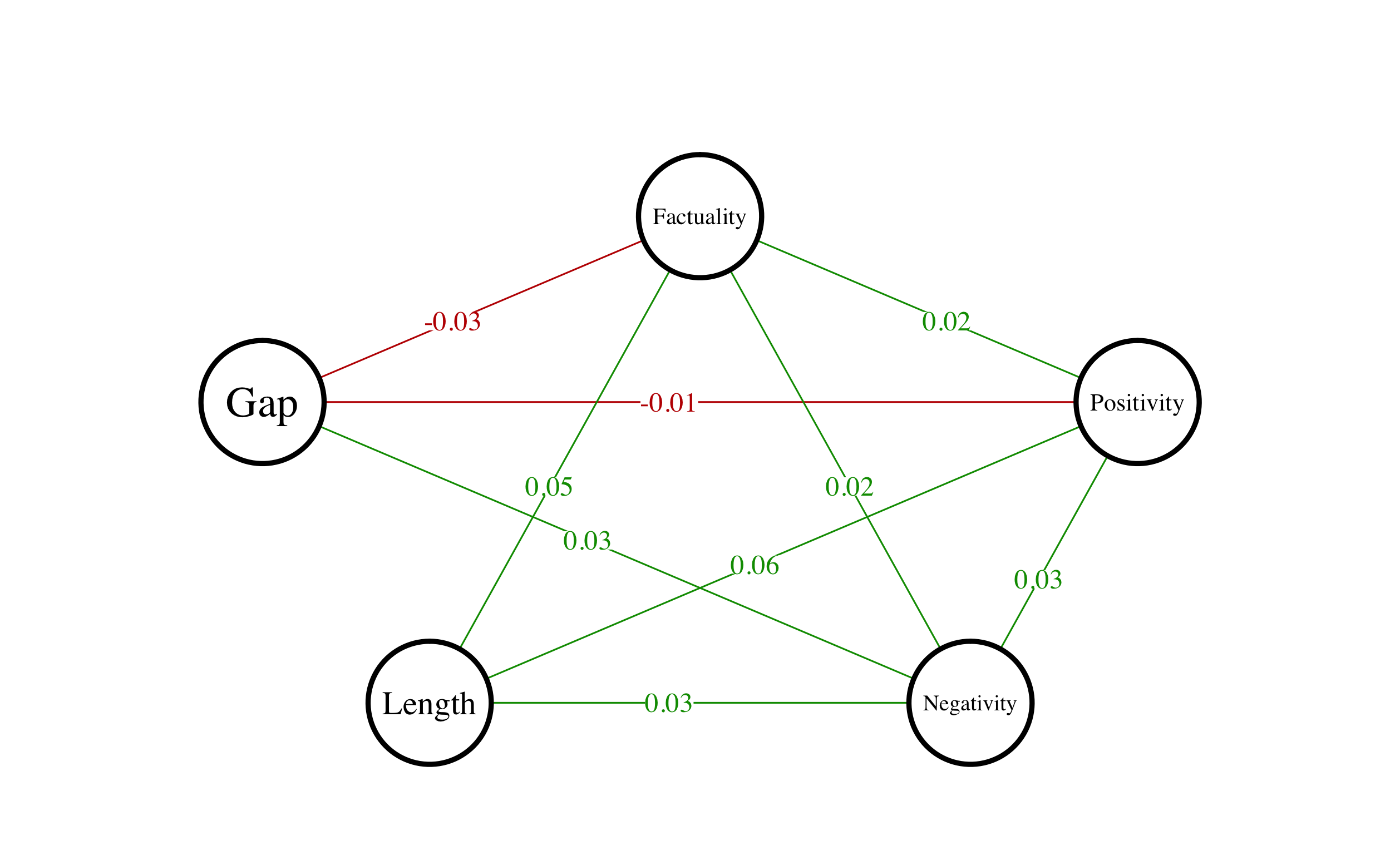


**Figure S1.** Contemporaneous temporal network of the multilevel vector autoregression model.
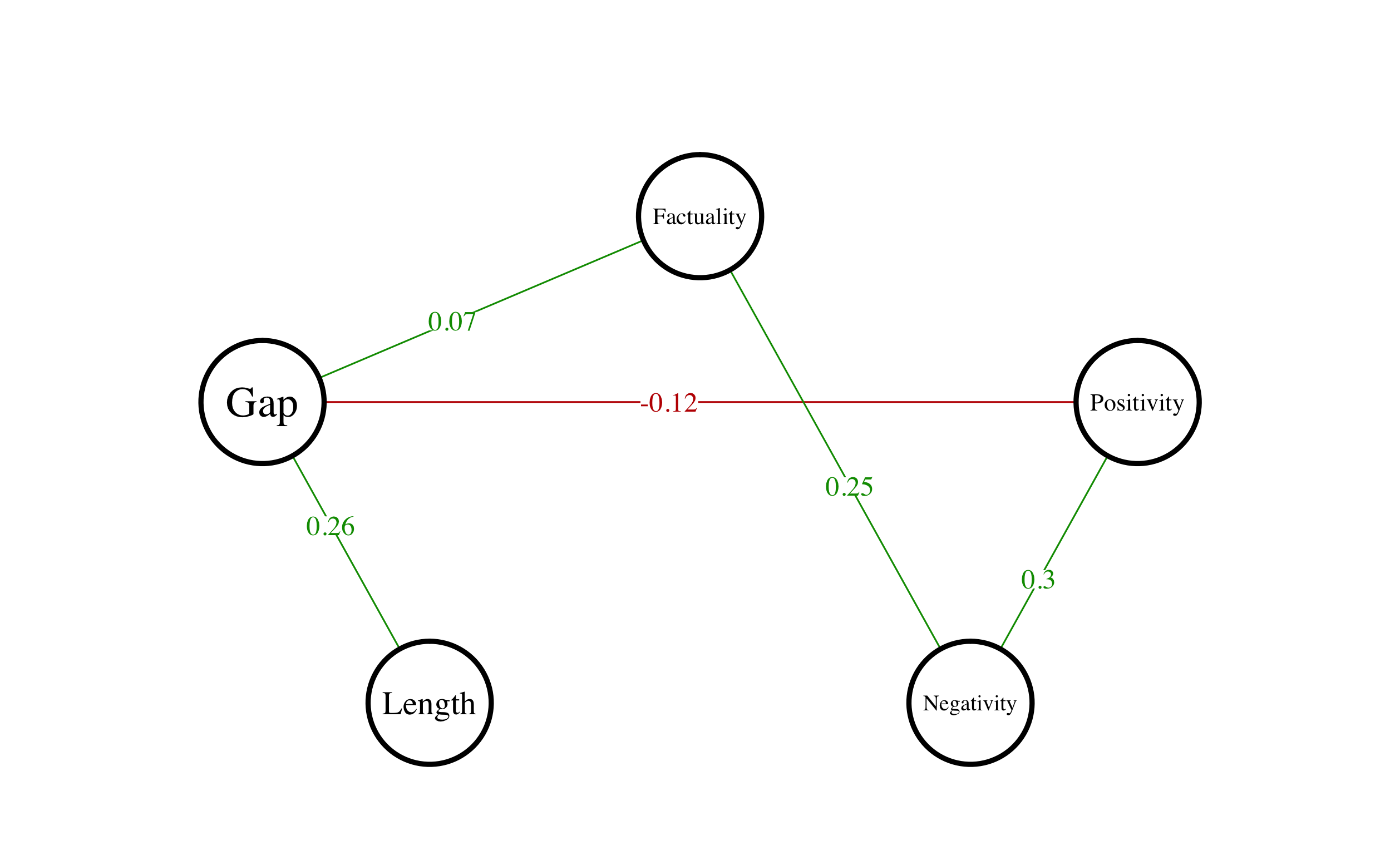


**Figure S2.** Between-person network of the multilevel vector autoregression model.

*Contemporaneous and Between-Person Effects.* Tables S1 and S2 shows the estimates of contemporaneous and between-person effects of the multilevel VAR model. Figures S1 and S2 graphically represent the effects. In these figures, a green edge represents a positive partial correlation and a red edge a negative partial correlation. Only the edges representing significant results are shown with the correlation labeled. The contemporaneous network in Figure S1 demonstrates how measures are correlated within the same sessions. For example, the green edge between positivity and length in this network indicates that sessions that contained more positive content tend to be longer. The between-person network in Figure S2 shows how measures are correlated at the participant level. For instance, the red edge between positivity and gap indicates that participants who consumed more positive content tend to have shorted gaps between sessions. While not directly related to the hypotheses tested in this study, estimates of the between-person effects are mostly consistent with our findings from the temporal effects network. The positive partial correlations between factuality and negativity, between positivity and negativity, between length and gap, and the negative partial correlation between positivity and gap are all consistent with the temporal effects between these measures.


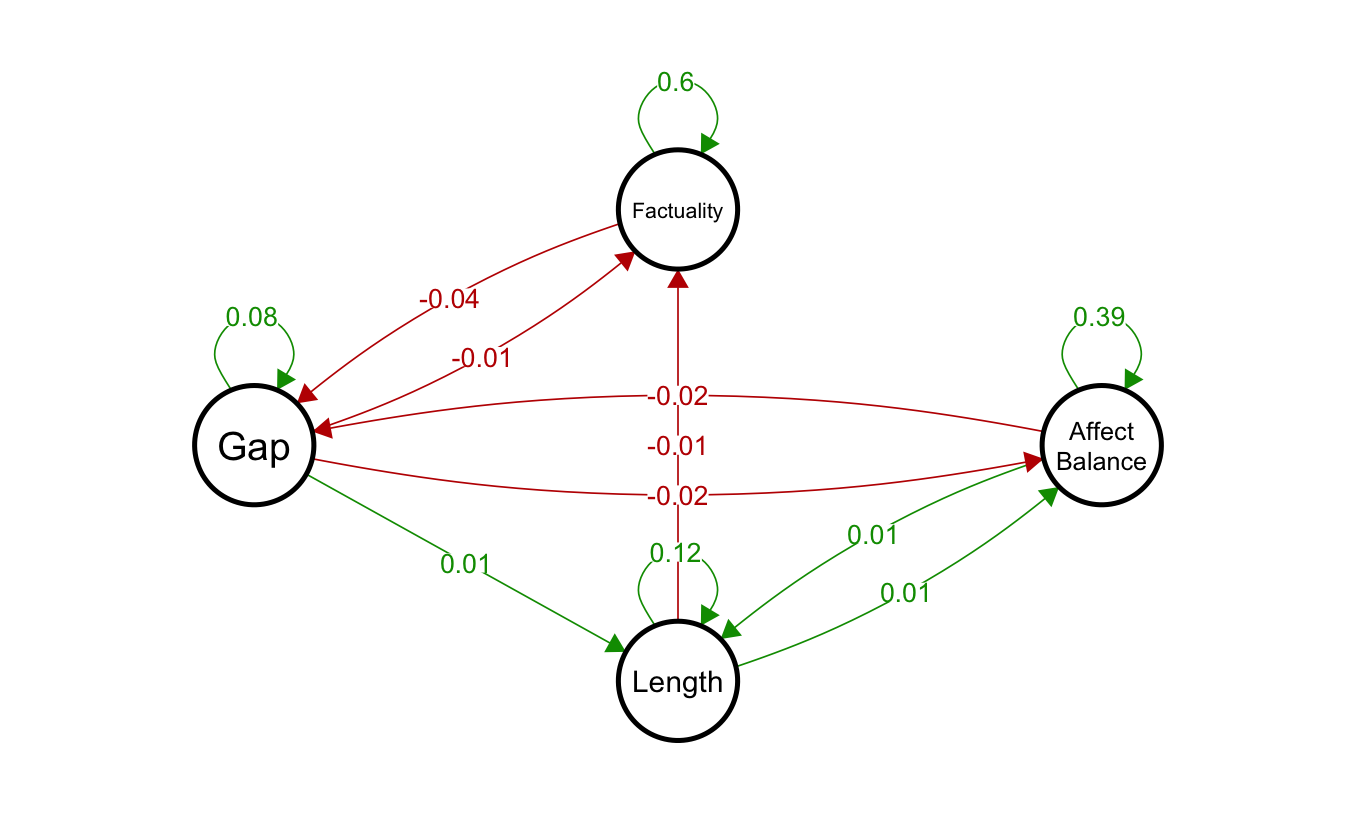


**Figure S3.** Temporal network of the affective balance multilevel vector autoregression model.

*Temporal effects of affect balance.* Figure S3 shows the temporal effects of a multilevel vector autoregression model of affective balance. Affective balance is computed as the value of positivity minus negativity. This model allowed us to examine how the relations differ when positivity and negativity are combined into a single variable. The lag-1 autoregression of the new affective balance variable (computed as positivity minus negativity) is of similar sign and size as the lag-1 autoregression of positivity and negativity variables (+.39 vs. +.42 and +.35). Other parts of the model were similar, including the relation between gap and length. However, whereas the model in the main text indicates there are relations between factuality and both positivity and negativity, there is no relation between factuality and affect balance in this model – perhaps because differential relations of the component parts cancel each other out within the unified affect balance variable.
